# On the Use of the “Radiometer” in Testing the Vision

**Published:** 1881-12

**Authors:** Lucien Howe


### On the Use of the "Radiometer" in Testing the Vision.

By Lucien Howe, M. D.

With a view to greater accuracy in our observations, it has occurred to me an improvement might be made in the manner of recording the vision of our patients. It is true that Snellen, Jaeger, Greene, and others, have furnished excellent tests in the form of types, but as yet we have no standard by which to measure the amount of light to be admitted upon them at the time of making our observations. We naturally understand that these test letters are to be viewed under such illumination as render them distinguishable at a given distance.

In this case we say the light is "sufficient," or "good," or "fair," and we express the vision by means of a fraction, the numerator of which represents the distance in feet at which the letters are placed, and the denominator shows which set of letters is seen. Thus $V=\frac{20}{20}$, means that at twenty feet (a distance from which rays enter the eye practically parallel) the patient sees type No. 20, and the size of this set of letters is such as to subtent only the angle of minimum vision, or, in other words, they are the smallest that can be seen at that distance by the normal eye. Such a formula, therefore, expresses perfect vision. Or, $V=\frac{20}{30}$, $V=\frac{20}{40}$, means that at 20 feet, only a type is seen which *should* be visible at 30 or at 40 feet. This formula is fully explained in every text book on diseases of the eye, and is too well known to require more than simple mention here.

Not always, however, is there a perfect eye at hand for purposes of comparison, and the employment of any indefinite and varying method is, to say the least, inexact and unscientific. For instance, the vision of a patient may be recorded as $\frac{20}{20}$ on a bright day, when the types are hung in the full glare of sunlight. whereas in diffused light it may be $\frac{20}{30}$, or with the questionably perfect illumination of a rainy day it may reach only $\frac{20}{50}$.

It seemed to me, therefore, that it would greatly aid the correctness of such observations if they were always made under the same conditions, and if we had some convenient photometer which would readily indicate some illumination which might be agreed upon as a standard. Such an instrument has been devised by the English physicist, Professor Crookes, and called by him a radiometer. It will be remembered that in the main it consists of two fine wires crossing each other at right angles, and bearing at the extremities pith disks, one side of which is blackened, and the reverse covered with tin foil. At the intersection of these wires they are balanced upon a needle point, and the whole is enclosed in a glass bulb, some two inches in diameter, from which bulb the air is exhausted. Now, when the light falls upon these disks, it causes them to revolve about the needle point with a rapidity which is practically proportional to its intensity, thus giving us a photometer. There is still much doubt as to whether it revolves from the action of those waves of motion which we call light, or by virtue of those also to which we give the name of electricity; but practically, for our purpose, this makes but little difference. It is true, also, that every radiometer revolves at a rate peculiar to itself under a given illumination, but we have the standard of direct sunlight with which to test each instrument and make our record accordingly.

For instance, the one I have been accustomed to use, makes about twenty revolutions per minute, when exposed to direct sunlight. If, however, it is under such an illumination as may justly be termed "good" or "fair," it revolves much more slowly. In observing a patient's vision, therefore, I regulate the amount of light, making it more on a dark, cloudy day, and lessening its intensity on a clear one to such a degree that the radiometer will make a certain number, say five, revolutions in a minute. When the same patient is seen again, I am able to judge precisely as to improvement or other changes, however slight they may be, which have taken place, and to be certain that the conclusions drawn are absolutely correct. During the short time that I have employed this method of observing the vision, it has proved so simple, so exact, and in general so satisfactory, as to seem worthy of mention.

